# Methylation and transcriptome analysis reveal lung adenocarcinoma-specific diagnostic biomarkers

**DOI:** 10.1186/s12967-019-2068-z

**Published:** 2019-09-27

**Authors:** Rui Li, Yi-E Yang, Yun-Hong Yin, Meng-Yu Zhang, Hao Li, Yi-Qing Qu

**Affiliations:** 1 Department of Respiratory and Critical Care Medicine, Qilu Hospital of Shandong University, Jinan, 250012 China; 2 Department of Clinical Laboratory, Qianfoshan Hospital of Shandong Province, Jinan, 250014 China

**Keywords:** Lung adenocarcinoma, Methylation-driven lncRNA, Methylation-driven mRNA, Biomarkers, Overall survival rate

## Abstract

**Background:**

DNA methylation can regulate the role of long noncoding RNAs (lncRNAs) in the development of lung adenocarcinoma (LUAD). The present study aimed to identify methylation-driven lncRNAs and mRNAs as biomarkers in the prognosis of LUAD using bioinformatics analysis.

**Methods:**

Differentially expressed RNAs were obtained using the edge R package from 535 LUAD tissues and 59 adjacent non-LUAD tissues. Differentially methylated genes were obtained using the limma R package from 475 LUAD tissues and 32 adjacent non-LUAD tissues. Methylation-driven mRNA and lncRNA were obtained using the MethylMix R package from 465 LUAD tissues with matched DNA methylation and RNA expression and 32 non-LUAD tissues with DNA methylation. Gene ontology and ConsensusPathDB pathway analysis were performed to identify functional enrichment of methylation-driven mRNAs. Univariate and multivariate Cox regression analyses were performed to identify the independent effect of each variable for predicting the prognosis of LUAD. Kaplan–Meier curve analysis of DNA methylation and gene expression might provide potential prognostic biomarkers for LUAD patients.

**Results:**

A total of 99 methylation-driven mRNAs and 17 methylation-driven lncRNAs were obtained. Univariate and multivariate Cox regression analysis showed that 6 lncRNAs (FOXE1, HOXB13-AS1_2, VMO1, HIST1H3F, AJ003147.8, ASXL3) were retrieved to construct a predictive model associated with overall survival in LUAD patients. Combined DNA methylation and gene expression survival analysis revealed that 4 lncRNAs (AC023824.1, AF186192.1, LINC01354 and WASIR2) and 8 mRNAs (S1PR1, CCDC181, F2RL1, EFS, KLHDC9, MPV17L, GKN2, ITPRIPL1) might act as independent biomarkers for the prognosis of LUAD.

**Conclusions:**

Methylation**-**driven lncRNA and mRNA contribute to the survival of LUAD, and 4 lncRNAs and 8 mRNAs might be potential biomarkers for the prognosis of LUAD.

## Background

Lung cancer is the leading cause of cancer-related death worldwide [1]. Lung adenocarcinoma (LUAD) accounts for 45–55% of non-small-cell lung cancer (NSCLC), with a 5-year overall survival rate of less than 15% [2]. Due to the heterogeneity of lung adenocarcinoma, it is still a great challenge to develop successful individual-based treatment [3]. Therefore, there is an urgently need to identify effective and promising biomarkers in predicting the prognosis of LUAD.

Genetic aberrant expression is crucial for cancer etiology, and the joint effect of both genetic and epigenetic changes facilitates the development of human cancer [4–6]. DNA methylation acts as the key element in epigenetic modifications and plays a significant role in the regulation of cellular functions and carcinogenesis [7–9]. Epigenetic modification, especially DNA methylation, can provide a novel horizon to explore new biomarkers in predicting the prognosis of cancer [10–14]. A large number of studies have demonstrated that DNA methylation can regulate the expression of lncRNA, and this phenomenon may be associated with the prognosis of lung cancer [15]. For instance, the lncRNA AFAP1-AS1 acts as an oncogene in NSCLC, while its expression is tightly regulated by DNA methylation, which might provide prognostic and diagnostic values for NSCLC patients [16].

MethylMix, an algorithm implemented in the R programming environment, identifies disease-specific hyper- and hypomethylated genes [17]. Currently, few studies on methylation-driven genes have been reported [18]. Recently, a study based on using MethylMix to explore methylation-driven genes for predicting the prognosis of LUAD was reported; however, they only obtained information about methylation-driven mRNA [19]. In this study, DNA methylation and RNA-Seq data were extracted from The Cancer Genome Atlas (TCGA) database, and we used the MethylMix R package to obtain LUAD-specific methylation-driven lncRNA sequences. Furthermore, a Cox survival predictive model with 6 lncRNAs was constructed to predict the diagnosis and prognosis of LUAD. Finally, the combined effect of DNA methylation and gene expression survival analysis was examined, which might provide a novel insight to explore methylation-driven lncRNA and mRNA for predicting the prognosis of LUAD.

## Methods

### Data retrieving and analyzing

Methylation and RNA-Seq expression data were retrieved from LUADs from the TCGA database. The methylation data were downloaded from 475 cancer tissues and 32 noncancer tissues from the Illumina Human methylation 450k platform. The RNA-Seq data (level 3), including mRNA and lncRNA expression, were retrieved from 535 cancer tissues and 59 noncancer tissues from the IlluminaHiSeq_RNASeq platform. First, on the basis of “limma R” packages in R with absolute fold change (log 2) > 0 and adjusting the false discovery rate (FDR) to a *P* value < 0.05, we obtained aberrant methylated genes. The methylation difference of the mean of the 3000 bp (base pair) sites upstream of the gene was analyzed to identify the differential level of methylation in the gene promoter [19–22]. We analyzed the differential level of methylation in the promoter of genes by using the limma R package [23]. Then, based on the “edge R” packages in R with absolute fold change (log 2) > 3 and adjusting the false discovery rate (FDR) to a *P* value < 0.01 to correct the statistical significance of multiple experiments, we retrieved differentially expressed mRNA and lncRNA. MethylMix is a kind of R statistical package for integrating DNA methylation data and RNA expression data to identify methylation-driven genes in kinds of cancers [17, 24–26]. Filtering or eliminating missing value genes is the preprocessing common step when running MethylMix R software [17]. In this present manuscript, we filtered or eliminated missing value genes and intersected DNA methylation data with RNA expression data for matching. Finally, there were a total of 465 LUAD samples with matched DNA methylation and RNA expression and 32 non-LUAD samples with DNA methylation data for entering the MethylMix R package. Then, calculated the correlation between DNA methylation level and RNA expression to find significantly negatively related genes, a beta mixture model was constructed for the degree of methylation of samples, Wilcoxon rank test was used to calculate differential methylation in LUAD and adjacent non-LUAD samples. Finally, the methylation-driven mRNA and lncRNA was obtained. Since the data were directly obtained from the TCGA database, no approval was required from the local ethics committee.

### Functional enrichment analysis of methylation-driven mRNA in LUAD

To determine the function represented in the methylation-driven mRNA, we used the Database for Annotation, Visualization and Integrated Discovery (DAVID) (http://david.abcc.ncifcrf.gov/) to perform a functional and enrichment analysis on the methylation-driven mRNA by using GO and ConsensusPathDB analysis. In the GO analysis, a *P* value of less than 0.05 was considered statistically significant. Furthermore, the GOCircle and GOChord plotting functions of the GOplot R package were used to allow data from expression analysis and data from functional annotation enrichment analysis. ConsensusPathDB (http://cpdb.molgen.mpg.de/) is an online software program that includes binary and complex signaling, gene regulatory and drug-target interactions, and biochemical pathways. *P *< 0.05 was considered statistically significant.

### Construction of a differentially methylated, lncRNA-related predictive model in LUAD

We identified the differentially methylated lncRNA associated with overall survival with *P *< 0.05 to act as prognostic methylation lncRNA candidates for multivariate Cox regression analysis. On the basis of the median risk score, LUAD patients were divided into two cohorts, high-risk cohorts and low-risk cohorts. Receiver operating curves were used to test the effect of the lncRNA signature (high risk vs low risk) on overall survival. We analyzed the receiver operating curve by calculating the area under the curve (AUC) under the binomial exact confidence interval to reveal prognostic biomarkers for predicting survival in LUAD.

### Combined methylation and gene expression survival analysis

To further explore the effect of methylation level and gene expression level of the same methylation-driven key gene on LUAD patient prognosis, we performed a combined methylation and gene expression survival analysis to identify potential methylation-driven mRNAs and methylation-driven lncRNAs for predicting the prognosis of LUAD patients. Therefore, a Kaplan–Meier curve was performed. *P *< 0.05 was regarded as statistically significant.

## Results

### Identification of methylation-driven mRNA and lncRNA in LUAD

**Fig. 1:**
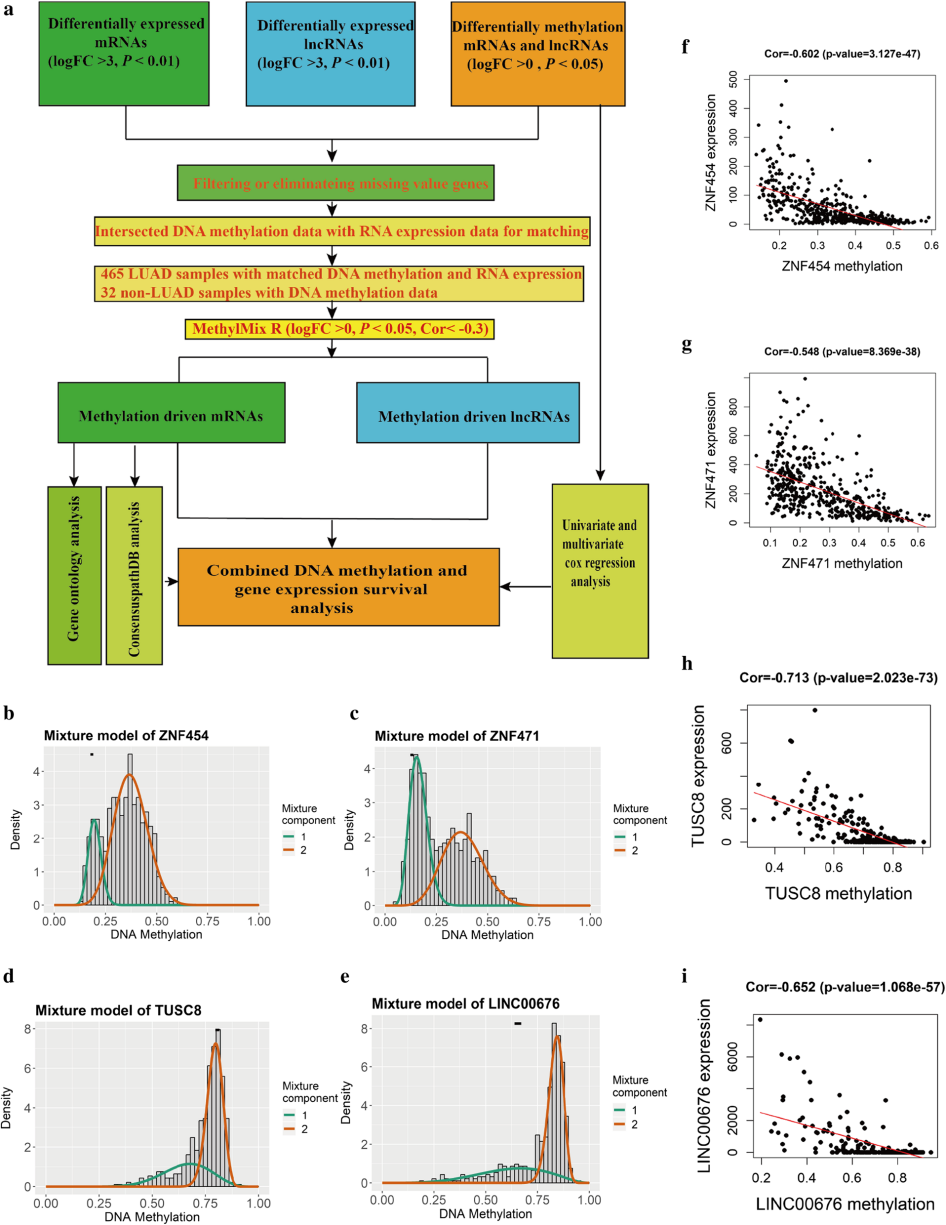
Identification of top hypermethylated and hypomethylated mRNAs and lncRNAs in LUAD. **a** A flow diagram of the exploration of methylation-driven mRNA and lncRNA in LUAD. **b**–**e** The methylation degree when comparing cancer patients to normal patients in LUAD. The red curve indicates the methylation degree from the cancer group, the green curve indicates the methylation degree from the normal group, and the black line above the figure is the distribution of methylation levels in normal patients. **f**–**i** The correlation between methylation and gene expression in methylation-driven mRNAs and lncRNAs

**Fig. 2:**
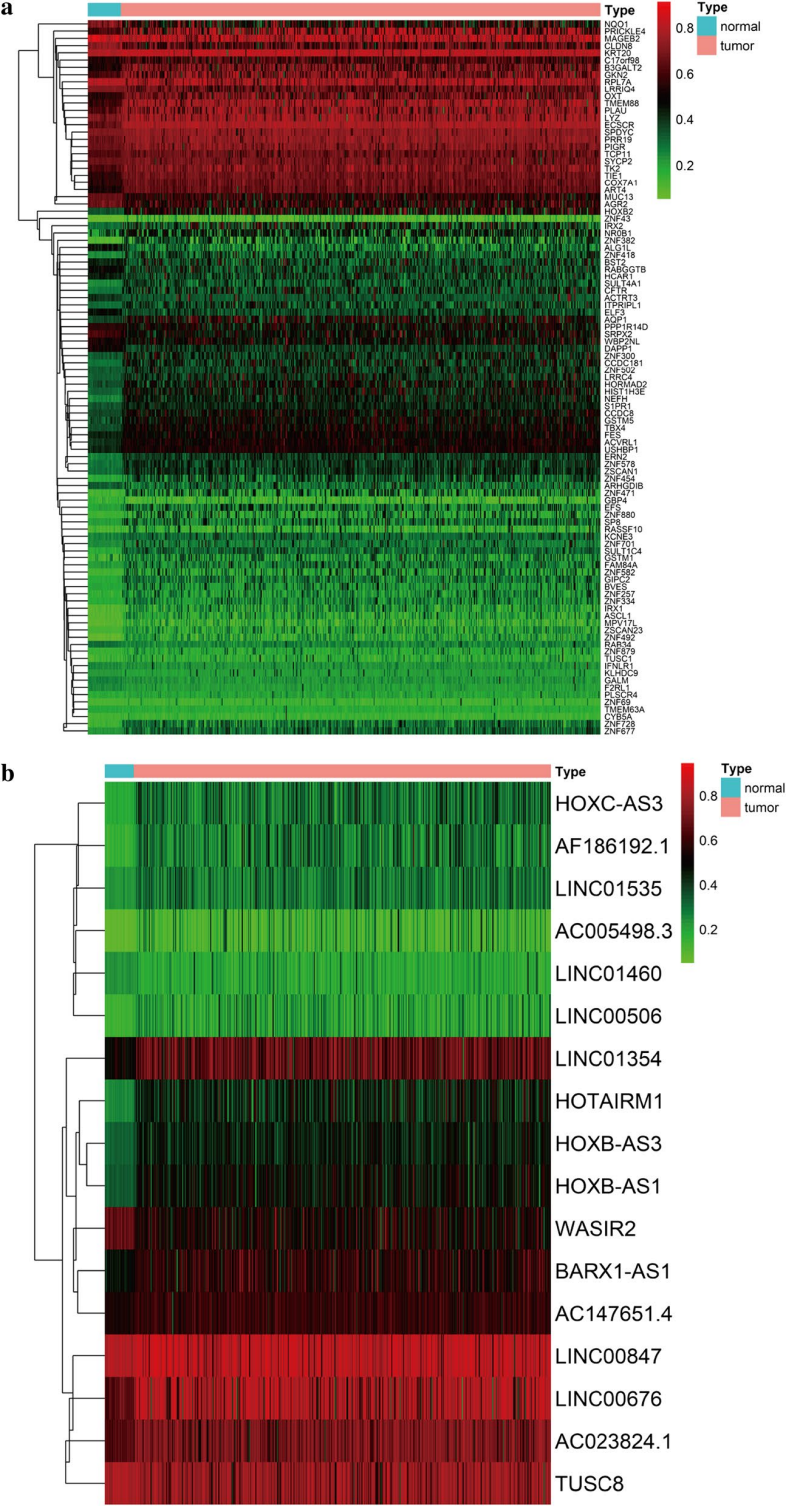
Heat map of methylation-driven mRNAs and lncRNAs in LUAD. **a** The hierarchical clustering heat map of LUAD-specific methylation-driven mRNAs. **b** The hierarchical clustering heat map of LUAD-specific methylation-driven lncRNAs. In the figure, red represents highly methylated genes and green represents low methylated genes between LUAD and adjacent tissues

A total of 99 mRNAs and 17 lncRNAs were identified to be associated with DNA methylation using MethylMix criteria. The methylation-driven mRNAs and IncRNAs are shown in Tables 1 and 2, respectively. We constructed a mixed model and performed a Wilcoxon rank test for determining differential methylation (logFC > 0, *P *< 0.05, Cor < − 0.3). Figure 1 shows that two methylation-driven mRNAs (ZNF454 and ZNF471) (Fig. 1f, g) and two methylation-driven lncRNAs (TUSC8 and LINC00676) (Fig. 1h, i) have significant negative correlations between methylation and gene expression levels. In Fig. 1, the distribution of the methylation degree shows that ZNF454 (Fig. 1b), ZNF471 (Fig. 1c), and LINC00676 (Fig. 1e) are hypermethylated in LUAD patients and hypomethylated in the normal group, while TUSC8 (Fig. 1d) is hypomethylated in LUAD patients and hypermethylated in the normal group. A heat map of methylation-driven mRNAs and lncRNAs is shown in Fig. 2a, b. A flow diagram of the exploration of methylation-driven mRNA and lncRNA in LUAD is shown in Fig. 1a.

**Table 1:** Methylation-driven mRNAs

mRNA	Normal mean	Tumor mean	logFC	*P* Value	Adjusted-P	Cor	Cor P-value
ECSCR	0.651549745	0.777875207	0.255663395	6.97E−21	1.03E−18	− 0.401694346	1.85E−19
TBX4	0.3491894	0.503087408	0.526799315	7.58E−21	1.12E−18	− 0.353891186	3.64E−15
TIE1	0.559830867	0.675047137	0.269997212	1.21E−20	1.79E−18	− 0.406629901	6.08E−20
NEFH	0.254444122	0.435542374	0.775464224	1.40E−20	2.07E−18	− 0.321798888	1.16E−12
ACVRL1	0.398975345	0.517535129	0.375357195	3.26E−20	4.82E−18	− 0.386877674	4.73E−18
USHBP1	0.419199887	0.525059456	0.324842471	3.63E−20	5.37E−18	− 0.323007247	9.42E−13
ERN2	0.264647925	0.387648278	0.550673914	3.92E−20	5.80E−18	− 0.450728621	1.20E−24
COX7A1	0.560388444	0.666059579	0.249224026	9.41E−20	1.39E−17	− 0.342569242	3.00E−14
SULT1C4	0.176980042	0.3113128	0.814778224	2.45E−19	3.62E−17	− 0.375338318	5.28E−17
ART4	0.531234199	0.652680788	0.297029548	2.53E−19	3.75E−17	− 0.425340294	7.44E−22
HIST1H3E	0.313889479	0.456138176	0.539214244	1.45E−18	2.15E−16	− 0.390470196	2.19E−18
ZNF492	0.077650427	0.196364234	1.33846642	1.18E−17	1.75E−15	− 0.378866342	2.55E−17
ZNF728	0.138731042	0.290955121	1.068506001	1.32E−17	1.95E−15	− 0.346356662	1.50E−14
S1PR1	0.33644215	0.424456445	0.33525806	4.34E−17	6.43E−15	− 0.375495078	5.11E−17
MUC13	0.657841786	0.551979674	− 0.253125507	4.44E−17	6.57E−15	− 0.402604202	1.51E−19
CCDC8	0.360671572	0.480500959	0.413853596	8.99E−17	1.33E−14	− 0.389064482	2.96E−18
ZNF578	0.281145557	0.394615614	0.489130794	1.12E−16	1.66E−14	− 0.459621578	1.11E−25
FES	0.424918825	0.49946057	0.233183531	2.74E−16	4.05E−14	− 0.370960412	1.29E−16
ASCL1	0.096716299	0.200703748	1.053236604	2.98E−16	4.41E−14	− 0.331763879	2.08E−13
ALG1L	0.461379719	0.287062126	− 0.684591589	3.14E−16	4.65E−14	− 0.466908378	1.49E−26
ELF3	0.462412193	0.359973363	− 0.361289285	4.47E−16	6.61E−14	− 0.45067724	1.22E−24
TMEM88	0.583625618	0.738189625	0.338948249	7.89E−16	1.17E−13	− 0.390308898	2.27E−18
GSTM5	0.356896303	0.481942079	0.433354815	1.13E−15	1.68E−13	− 0.353462769	3.95E−15
IRX1	0.098629887	0.220552417	1.161024783	1.21E−15	1.79E−13	− 0.344157687	2.24E−14
ZNF454	0.183616121	0.338501411	0.88246912	1.44E−15	2.13E−13	− 0.602235321	3.13E−47
TK2	0.615291641	0.702921916	0.192094042	1.71E−15	2.52E−13	− 0.365352213	3.94E−16
ZSCAN1	0.289768404	0.400355348	0.466380783	2.83E−15	4.19E−13	− 0.397488998	4.73E−19
ZNF677	0.220497196	0.315626597	0.517458471	2.86E−15	4.23E−13	− 0.527352048	1.21E−34
ZNF582	0.130372053	0.239679107	0.878469505	5.74E−15	8.49E−13	− 0.558968642	1.43E−39
DAPP1	0.510432574	0.394016678	− 0.373463704	1.29E−14	1.91E−12	− 0.490584559	1.57E−29
SRPX2	0.600591335	0.480798168	− 0.32095226	1.42E−14	2.10E−12	− 0.383430149	9.83E−18
CCDC181	0.283605585	0.41823899	0.560441617	3.23E−14	4.78E−12	− 0.319044754	1.84E−12
SULT4A1	0.24652482	0.348099193	0.497765567	7.69E−14	1.14E−11	− 0.358208198	1.59E−15
LRRC4	0.321370351	0.418618502	0.38139924	1.26E−13	1.87E−11	− 0.421045183	2.10E−21
ZSCAN23	0.12251013	0.209336095	0.772920046	1.57E−13	2.32E−11	− 0.362584137	6.80E−16
F2RL1	0.242790645	0.208656331	− 0.218584138	1.74E−13	2.57E−11	− 0.35941342	1.26E−15
ZNF334	0.17466699	0.256829933	0.556206371	2.31E−13	3.43E−11	− 0.459451082	1.16E−25
ZNF471	0.130738051	0.279080546	1.094002465	5.46E−13	8.07E−11	− 0.548011494	8.37E−38
HOXB2	0.34354942	0.513088034	0.578688733	6.70E−13	9.91E−11	− 0.491155695	1.32E−29
PRR19	0.666407262	0.728756031	0.129031796	9.23E−13	1.37E−10	− 0.420836129	2.20E−21
AGR2	0.669953934	0.554274136	− 0.273462209	1.68E−12	2.49E−10	− 0.51205684	1.93E−32
NQO1	0.702158099	0.525979398	− 0.416789616	2.08E−12	3.08E−10	− 0.425444078	7.26E−22
GIPC2	0.179310415	0.281572165	0.651045433	3.52E−12	5.21E−10	− 0.36721046	2.73E−16
OXT	0.553265288	0.656785573	0.247451026	4.61E−12	6.83E−10	− 0.519344744	1.77E−33
B3GALT2	0.534256964	0.658626901	0.301927631	6.78E−12	1.00E−09	− 0.477778193	6.83E−28
EFS	0.197613625	0.297157242	0.588544115	1.42E−11	2.10E−09	− 0.391422631	1.78E−18
RAB34	0.295528869	0.254270265	− 0.21693631	2.05E−11	3.03E−09	− 0.424411453	9.32E−22
ACTRT3	0.391186063	0.353188058	− 0.147418408	5.54E−11	8.21E−09	− 0.329154572	3.27E−13
CLDN8	0.727830255	0.618464903	− 0.234910297	9.70E−11	1.44E−08	− 0.328690081	3.55E−13
AQP1	0.366569984	0.501482299	0.452110121	2.34E−10	3.46E−08	− 0.597245937	2.74E−46
SP8	0.202841356	0.297916967	0.554558466	2.69E−10	3.98E−08	− 0.317009628	2.58E−12
HORMAD2	0.350476203	0.461146819	0.395909658	3.14E−10	4.65E−08	− 0.457907737	1.76E−25
GALM	0.255948625	0.226747119	− 0.174770041	6.86E−10	1.02E−07	− 0.356239606	2.33E−15
RABGGTB	0.47835678	0.385164319	− 0.312612985	6.89E−10	1.02E−07	− 0.3330019	1.67E−13
KCNE3	0.256671142	0.314316199	0.292295525	7.52E−10	1.11E−07	− 0.350037722	7.53E−15
ZNF879	0.147629821	0.2000367	0.438280535	1.01E−09	1.50E−07	− 0.453856587	5.23E−25
ZNF257	0.157589016	0.245609973	0.640202155	1.28E−09	1.89E−07	− 0.359120932	1.34E−15
ZNF382	0.101492	0.295488981	1.541738316	1.35E−09	1.99E−07	− 0.391566521	1.73E−18
GSTM1	0.156396431	0.253910031	0.699109797	2.76E−09	4.08E−07	− 0.563212162	2.84E−40
PLAU	0.62150196	0.702145935	0.176011973	4.00E−09	5.92E−07	− 0.570934425	1.41E−41
PIGR	0.667952501	0.708575582	0.085176236	5.52E−09	8.16E−07	− 0.658618149	3.68E−59
CFTR	0.282943087	0.394972666	0.481240926	9.95E−09	1.47E−06	− 0.42150843	1.88E−21
SPDYC	0.704147468	0.724837107	0.041779213	1.76E−08	2.60E−06	− 0.47588325	1.18E−27
ZNF418	0.242511045	0.347906619	0.520649675	2.19E−08	3.23E−06	− 0.60435923	1.23E−47
KLHDC9	0.218223326	0.21602453	− 0.014610182	2.63E−08	3.90E−06	− 0.324984031	6.72E−13
ZNF69	0.117564379	0.142619404	0.278719274	2.66E−08	3.94E−06	− 0.321104504	1.30E−12
FAM84A	0.212175332	0.283041565	0.415757	3.53E−08	5.23E−06	− 0.335916441	9.96E−14
PPP1R14D	0.554947827	0.482097369	− 0.203027589	6.64E−08	9.82E−06	− 0.560710402	7.40E−40
TCP11	0.624104627	0.659912943	0.080487807	7.18E−08	1.06E−05	− 0.651746879	1.43E−57
ZNF300	0.328409005	0.415250076	0.338486746	9.38E−08	1.39E−05	− 0.472110183	3.46E−27
MPV17L	0.092385716	0.163253978	0.821376431	1.86E−07	2.75E−05	− 0.441792534	1.23E−23
KRT20	0.790374237	0.804070021	0.02478522	2.92E−07	4.33E−05	− 0.571800235	1.00E−41
GKN2	0.599299248	0.671212935	0.163493955	4.56E−07	6.76E−05	− 0.340697654	4.22E−14
ZNF502	0.329208879	0.402697069	0.290691725	6.68E−07	9.88E−05	− 0.663271171	2.91E−60
C17orf98	0.545496557	0.602566194	0.143549644	1.03E−06	0.000151843	− 0.529865333	5.12E−35
ZNF880	0.140875018	0.238786926	0.76130805	1.04E−06	0.000154315	− 0.560715099	7.38E−40
ZNF701	0.240700225	0.290476188	0.271182411	1.55E−06	0.000229592	− 0.408643207	3.83E−20
NR0B1	0.283614274	0.392651776	0.469320279	1.59E−06	0.000235507	− 0.320709703	1.39E−12
ZNF43	0.067179039	0.115788942	0.78541442	1.79E−06	0.000265629	− 0.375124186	5.52E−17
HCAR1	0.43400231	0.376162376	− 0.206347167	1.93E−06	0.000285658	− 0.401199574	2.07E−19
IRX2	0.289887257	0.40281496	0.474625351	3.59E−06	0.000530859	− 0.539421825	1.83E−36
TMEM63A	0.188744901	0.177446694	− 0.089051973	4.10E−06	0.000607535	− 0.345096982	1.89E−14
ITPRIPL1	0.294502802	0.374069204	0.345023839	4.14E−06	0.000613146	− 0.44960488	1.61E−24
LYZ	0.699231698	0.733682794	0.069385864	4.77E−06	0.000705417	− 0.424840466	8.40E−22
IFNLR1	0.246226199	0.232227751	− 0.084443891	9.60E−06	0.001421224	− 0.420673159	2.29E−21
TUSC1	0.13659181	0.18296341	0.421684184	9.60E−06	0.001421229	− 0.395587597	7.19E−19
MAGEB2	0.842482835	0.77366049	− 0.122946694	1.08E−05	0.001598591	− 0.352008819	5.20E−15
BVES	0.191406638	0.252664551	0.400582407	1.12E−05	0.001655808	− 0.321449081	1.23E−12
LRRIQ4	0.704379076	0.645150221	− 0.126716926	1.21E−05	0.001791741	− 0.38912631	2.93E−18
RASSF10	0.120420032	0.181039038	0.588225416	5.00E−05	0.007400364	− 0.328252794	3.83E−13
PRICKLE4	0.605330662	0.686379771	0.181283603	8.15E−05	0.012068682	− 0.318883347	1.89E−12
SYCP2	0.665037997	0.675303689	0.022099668	0.000123074	0.018214969	− 0.655943521	1.55E−58
WBP2NL	0.524579367	0.489275744	− 0.1005133	0.000184004	0.027232614	− 0.343246032	2.65E−14
BST2	0.422881206	0.380549863	− 0.152166944	0.000187292	0.027719241	− 0.589617095	7.07E−45
PLSCR4	0.17571366	0.196664014	0.162506649	0.000241916	0.035803514	− 0.365149084	4.10E−16
GBP4	0.149941894	0.137445221	− 0.125546784	0.000249246	0.036888372	− 0.316476516	2.82E−12
RPL7A	0.801995847	0.754500966	− 0.088072019	0.000275207	0.040730644	− 0.319271372	1.77E−12
ARHGDIB	0.327607353	0.313354218	− 0.064173323	0.000282085	0.04174863	− 0.552243	1.77E−38
CYB5A	0.127300153	0.141309476	0.150624055	0.000302943	0.044835593	− 0.371106098	1.25E−16

**Table 2:** Methylation-driven lncRNAs

lncRNA	Normal mean	Tumor mean	logFC	P-value	Adjusted-P	Cor	Cor P-value
HOTAIRM1	0.250463518	0.44338384	0.823955709	2.68E−19	6.44E−18	− 0.326878646	4.85E−13
HOXB-AS3	0.319686633	0.449195438	0.490684853	5.50E−18	1.32E−16	− 0.412913672	1.43E−20
HOXB-AS1	0.335041907	0.484121893	0.531028779	1.17E−17	2.80E−16	− 0.459143798	1.26E−25
AF186192.1	0.153517115	0.289865274	0.916983002	1.36E−16	3.26E−15	− 0.432033057	1.44E−22
WASIR2	0.67568	0.534227796	− 0.33888511	3.03E−16	7.26E−15	− 0.38041164	1.85E−17
HOXC-AS3	0.180457648	0.304425709	0.754429909	3.10E−15	7.45E−14	− 0.33684433	8.44E−14
LINC01354	0.507119955	0.656977352	0.373516594	2.20E−14	5.28E−13	− 0.491825621	1.08E−29
BARX1-AS1	0.469474928	0.571905661	0.284729072	1.61E−13	3.87E−12	− 0.485400012	7.36E−29
AC005498.3	0.077393911	0.150762843	0.96198894	9.66E−11	2.32E−09	− 0.347615295	1.18E−14
AC147651.4	0.545827883	0.59134957	0.115565122	9.66E−11	2.32E−09	− 0.35424262	3.41E−15
LINC00676	0.65637148	0.770329464	0.230963053	2.88E−10	6.92E−09	− 0.652305415	1.07E−57
LINC01460	0.232370826	0.19315723	− 0.266653276	4.57E−09	1.10E−07	− 0.364997272	4.23E−16
AC023824.1	0.646522945	0.703429803	0.121704887	7.06E−09	1.69E−07	− 0.365841735	3.58E−16
LINC01535	0.227839948	0.292001828	0.357956684	4.99E−07	1.20E−05	− 0.371976592	1.05E−16
LINC00506	0.141670097	0.203560361	0.522921383	3.03E−05	0.000726506	− 0.330461443	2.61E−13
TUSC8	0.806078873	0.749074143	− 0.105812488	9.91E−05	0.002378923	− 0.713080072	2.02E−73
LINC00847	0.820916916	0.824558907	0.006386348	0.001130654	0.027135699	− 0.35046797	6.95E−15

### Enrichment analysis of methylation-driven mRNAs in LUAD

**Fig. 3:**
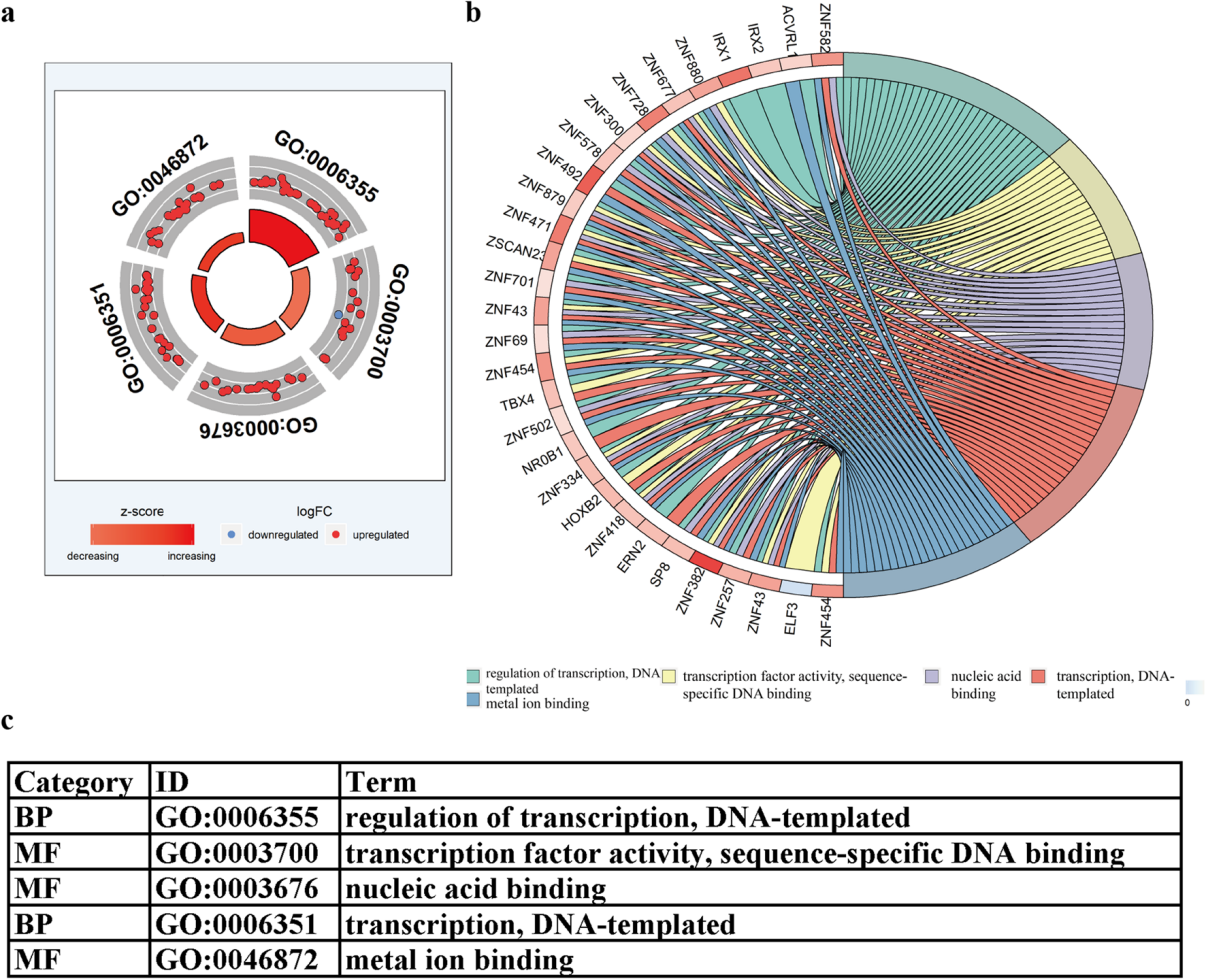
Functional enrichment analysis of methylation-driven mRNAs in LUAD. **a** The outer circle represents the expression (logFC) of methylation-driven mRNAs in each enriched GO (gene ontology) term: red dots on each GO term indicate upregulated methylation-driven mRNAs and blue dots indicate downregulated methylation-driven mRNAs. The inner circle indicates the significance of GO terms (log10-adjusted *P* values). **b** The circle indicates the correlation between the top 30 methylation-driven mRNAs and their gene ontology terms. **c** The distribution of the methylation-driven mRNAs in significant GO terms

**Fig. 4:**
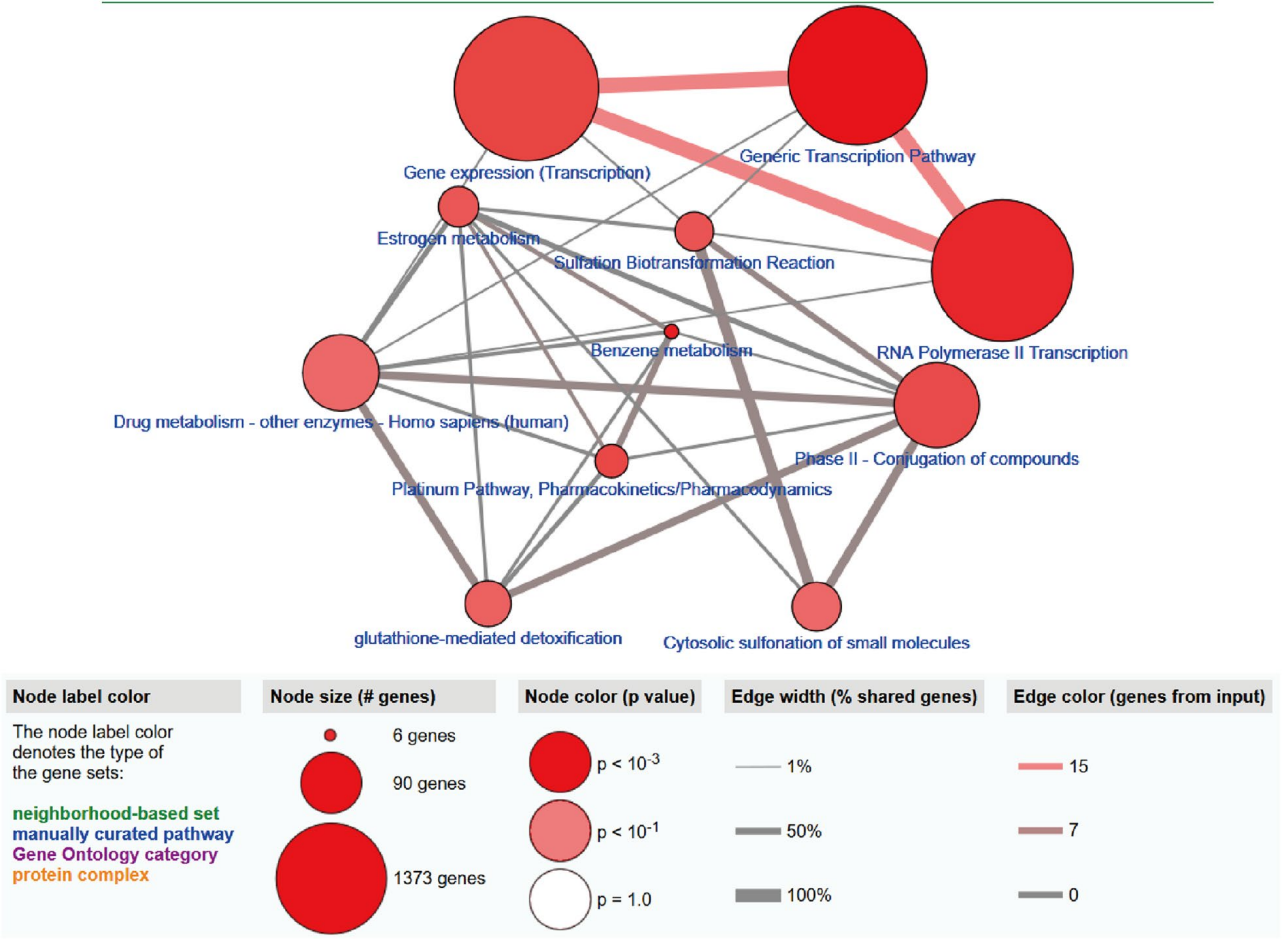
Pathway analysis of methylation-driven mRNAs in LUAD. The red circles indicate the number of methylation-driven mRNAs on each pathway. The line between the two red circles represents the ratio of methylation-driven mRNAs present in the common genes of the two pathways; the thicker the line, the more common methylation-driven mRNAs are represented

Gene ontology analysis showed that there were 5 GO terms (regulation of transcription, DNA-templated; transcription factor activity, sequence-specific DNA binding; nucleic acid binding; transcription, DNA-templated; metal ion binding) with significant differences (*P *< 0.05), and the highest GO biological process was “GO:0006355 regulation of transcription, DNA-templated” (Fig. 3a, c). The GOChord plot shows the top 30 methylation-driven mRNAs with their related GO terms (Fig. 3b). Figure 4 shows that 11 pathways (Generic Transcription Pathway, Benzene metabolism, RNA Polymerase II Transcription, Gene expression (Transcription), Platinum Pathway, Pharmacokinetics/Pharmacodynamics, Phase II—Conjugation of compounds, Sulfation Biotransformation Reaction, Estrogen metabolism, Glutathione-mediated detoxification, Cytosolic sulfonation of small molecules, Drug metabolism—other enzymes—Homo sapiens (human)) were considered statistically significant (*P *< 0.05). Furthermore, pathway analysis showed that the methylation-driven mRNAs were most enriched in the Generic Transcription Pathway, RNA Polymerase II Transcription and Gene expression (Transcription) pathways (*P *< 0.01) (Fig. 4). The pathway analysis is shown in Table 3.

**Table 3:** Pathway analysis

ID	Pathway	Count	P-value	q-value
R-HSA-212436	Generic transcription pathway	15	0.000169248	0.01031453
WP3891	Benzene metabolism	2	0.000338181	0.01031453
R-HSA-73857	RNA polymerase II transcription	15	0.000564162	0.011471298
R-HSA-74160	Gene expression (transcription)	15	0.001668004	0.020896371
PA150642262	Platinum pathway, Pharmacokinetics/pharmacodynamics	2	0.001720726	0.020896371
R-HSA-156580	Phase II—conjugation of compounds	4	0.002055381	0.020896371
WP692	Sulfation biotransformation reaction	2	0.002963269	0.025340686
WP697	Estrogen metabolism	2	0.003323369	0.025340686
PWY-4061	Glutathione-mediated detoxification	2	0.005884846	0.035982304
R-HSA-156584	Cytosolic sulfonation of small molecules	2	0.006376838	0.035982304
path:hsa00983	Drug metabolism—other enzymes—Homo sapiens (human)	3	0.006488612	0.035982304

### Construction of a predictive model of six differentially methylated lncRNAs in LUAD

Univariate Cox regression analysis was performed first to identify the prognosis associated with differentially methylated genes in LUAD, incorporating 10 methylation genes that were conspicuously associated with overall survival (*P *< 0.05). Next, multivariate Cox regression was used and showed that six lncRNAs were eventually selected to construct a predictive model. We used the linear combination of the expression of the 6 lncRNAs to construct the predictive model. The relative coefficients weighted in the multivariate Cox regression were as follows: survival risk score = (3.0040 × expression value of FOXE1 + 1.0226 × expression value of HOXB13-AS1_2 + 1.0540 × expression value of VMO1 + 1.0050 × expression value of HIST1H3F + (− 3.0925) × expression value of AJ003147.8 + 1.4791 × expression value of ASXL3). The multivariate Cox analysis is shown in Table 4.

**Table 4:** Multivariate Cox regression analysis of 6 lncRNAs associated with overall survival in LUAD patients

	coef	exp(coef)	se(coef)	z	P
FOXE1	3.004	20.1665	1.018	2.95	0.0032
HOXB13-AS1_2	1.0226	2.7804	0.5657	1.81	0.0706
VMO1	1.054	2.869	0.5992	1.76	0.0786
HIST1H3F	1.005	2.7319	0.3872	2.6	0.0094
AJ003147.8	− 3.0925	0.0454	0.6695	− 4.62	3.80E−06
ASXL3	1.4791	4.3888	0.7969	1.86	0.0635

### Risk groupings and ROC curve analysis

**Fig. 5:**
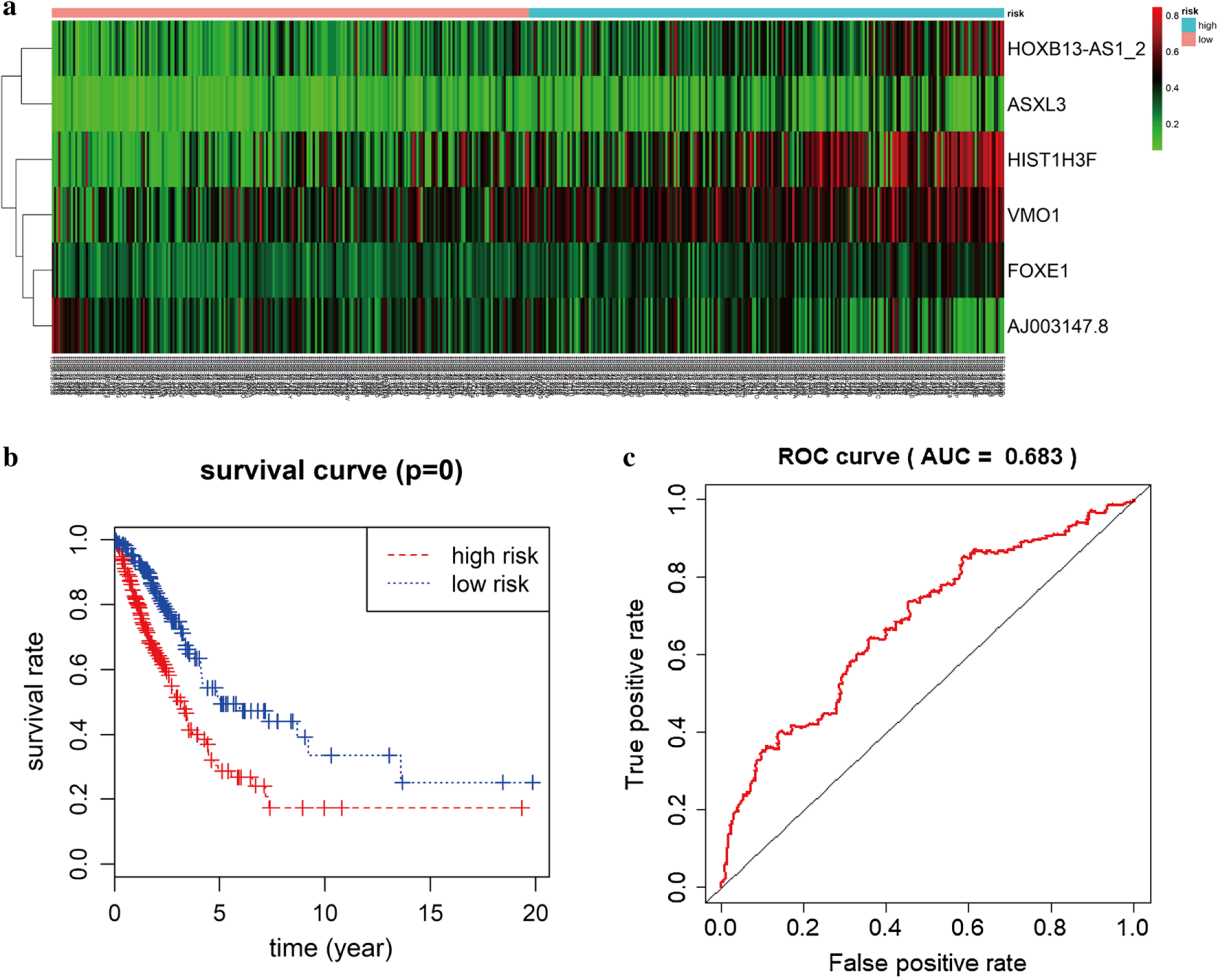
Prognostic value of 6-methylation lncRNAs in LUAD. **a** A risk heat map established from 6 lncRNAs from 449 LUAD patients. **b** Kaplan–Meier curve analysis for OS (overall survival) of LUAD patients using the 6 lncRNA signatures. **c** ROC curve analysis of the prognostic 6-lncRNA signature

As shown in the heat map, the expression of six prognostic methylation genes was profiled (Fig. 5a). Based on the median risk scores, a total of 449 samples of complete survival information were divided into a high-risk group (n = 224) and a low-risk group (n = 225). We used the Kaplan–Meier curve with a log-rank statistical examination to perform survival analysis. As shown in Fig. 5b, patients in the low-risk group had conspicuously better overall survival than those in the high-risk group (Fig. 5b). The receiver operating characteristic (ROC) curve was analyzed to test the influence on the 6-lncRNA signature associated with overall survival in LUAD (Fig. 5c).

### Combined methylation and gene expression survival analysis in LUAD

**Fig. 6:**
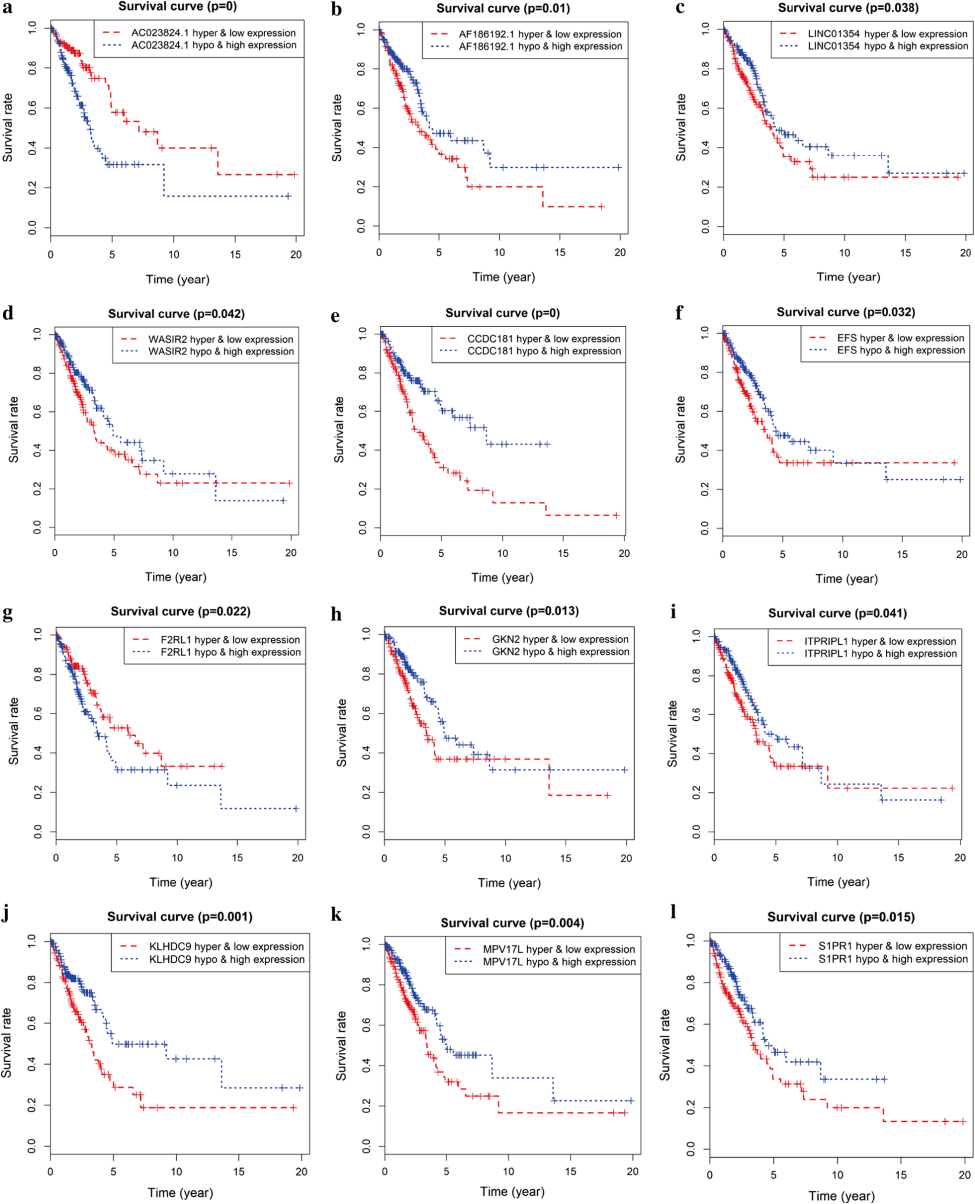
The combined methylation and gene expression data survival analysis in LUAD. **a**–**d** Kaplan–Meier curve analysis of four lncRNAs in LUAD. **e**–**l** Kaplan–Meier curve analysis of eight mRNAs in LUAD

The combined Kaplan–Meier curve analysis revealed that the combination of methylation and expression of lncRNAs AC023824.1, AF186192.1, LINC01354 and WASIR2 had a conspicuous correlation with the prognosis of LUAD patients **(**Fig. 6a–d**)**. The hypermethylation and low-expression survival rate of AC023824.1 was high, while the hypermethylation and low-expression survival rate of AF186192.1, LINC01354 and WASIR2 were low. The combined Kaplan–Meier curve analysis showed that the combination of methylation and mRNA expression of mRNAs CCDC181, EFS, F2RL1, GKN2, ITPRIPL1, KLHDC9, MPV17L, and S1PR1 were associated with overall survival of LUAD (*P *< 0.05) (Fig. 6e–l**)**. The hypermethylation and low-expression survival rate of F2RL1 was high. However, the hypermethylation and low-expression survival rates of EFS, CCDC181, GKN2, ITPRIPL1, KLHDC9, MPV17L, and S1PR1 were low (Fig. 6).

## Discussion

In recent years, with the increasing numbers of advanced diagnoses and poor prognoses in lung adenocarcinoma, it is pivotal to find more effective prognostic biomarkers to predict survival in LUAD. LncRNA-related studies have attracted the attention of various cancer fields. Accumulating studies show that cancer-related lncRNAs may serve as diagnostic or predictive biomarkers of cancer and have a significant effect on the therapeutic treatment of cancer [27]. Emerging evidence shows that studies on the molecular mechanisms and prognostic biomarkers of LUAD associated with methylation-driven lncRNA and mRNA are still lacking.

In recent years, epigenetic alterations in DNA methylation, noncoding RNA expression, chromatin modeling and post-transcriptional regulators have been found to play significant roles in the regulation and development of lung cancer pathogenesis [28–32]. Some studies have shown that epigenetic changes in DNA methylation cause changes in the expression of lncRNA, which might provide a novel insight to explore new biomarkers for predicting the prognosis of human cancer [33–36]. For instance, analysis of microarray data on gene expression and methylation showed that the expressions of lncRNAs LOC146880 and ENST00000439577 were regulated by DNA methylation, which might provide a new horizon to predict the diagnosis and prognosis of NSCLC [37]. Lu et al. indicated that MEG3 is significantly downregulated in NSCLC tissues that could be affected by DNA methylation [38]. Previous studies have shown that survival-associated, methylation-driven lncRNAs might serve as novel prognostic biomarkers for predicting the prognosis of LUAD.

Recent studies have shown that the roles of lncRNA in tumorigenesis and metastasis can indicate that lncRNA may function as a novel biomarker for the diagnosis and prognosis of cancer [39–44]. LncRNA TUBA4B has been reported to serve as a new predictor for prognosis and modulate cell viability in non-small-cell lung cancer [45]. lncRNA AFAP1-AS1 may act as an oncogenic to facilitate the migration of non-small-cell lung cancer (NSCLC) [16]. Long noncoding RNA ANRIL acts as an oncogene by silencing KLF2 and P21 expression to promote the development of NSCLC [46]. LncRNA PANDAR acts as a cancer suppressor gene by regulating Bcl-2 to affect cell apoptosis in NSCLC [47].

In the present study, we retrieved methylation and lncRNA and mRNA expression from the TCGA database by using bioinformatics analysis and obtained methylation-driven lncRNAs and mRNAs to predict the prognosis of LUAD. First, we obtained differentially expressed methylation and lncRNA and mRNA using the MethylMix R package to obtain methylation-driven lncRNA and mRNA. Functional enrichment analysis was performed to analyze the methylation-driven mRNA to identify its biological functions in the regulation and development of LUAD. Furthermore, univariate and multivariate Cox regressions were performed to construct a predictive model for predicting the prognosis of LUAD. Finally, a combined methylation and lncRNA expression survival analysis was carried out, which might provide novel insight to predict the diagnosis and prognosis of LUAD.

In the present study, we combined methylation and lncRNA and mRNA expression data with survival analysis to identify 4 lncRNAs and 8 mRNAs that act as independent prognostic factors for predicting the diagnosis and prognosis of LUAD. LINC01354 acts as a ceRNA to predict the early diagnosis and prognosis of colorectal cancer [48]. The combined survival analysis showed that the low expression of AC023824.1 with hypermethylation, compared to the high expression of AC023824.1 with hypomethylation, had a higher survival rate. (*P* = 0). The combined hypermethylation and low-expression survival rate of AF186192.1 was lower than the hypomethylation and high-expression survival rate of AF186192.1. (*P* = 0.01). The low expression and hypermethylation survival rate of LINC01354 was low. (*P* = 0.038). The high expression and hypomethylation survival rate of WASIR2 was high. The survival analysis showed that lncRNA AF186192.1, LINC01354 and WASIR2 might act as cancer suppressor genes regulated by DNA methylation to play significant roles in predicting the prognosis of LUAD. LncRNA AC023824.1 might act as an oncogene regulated by DNA methylation to have a pivotal effect on predicting the prognosis of LUAD. The survival rate of hypermethylation and low expression of CCDC181, EFS, GKN2, ITPRIPL1, KLHDC9, MPV17L, and S1PR1 were low. However, the hypermethylation and low-expression of the survival rate of F2RL1 was high. F2RL1 might act as an oncogene for predicting the prognosis of LUAD. Previous studies have shown that CCDC181, KLHDC9, and S1PR1 act as methylation-driven genes to reveal prognostic biomarkers in LUAD [19]. GKN2 may contribute to the homeostasis of gastric epithelial cells by inhibiting GKN1 activity [49]. F2RL1 may act as novel acute myeloid leukemia subsets that are meaning for treatment guidance [50]. MPV17L acts as a unique interacting protein and regulator of HtrA2 protease, mediating antioxidant and antiapoptotic functions in mitochondria [51]. Compared with previous studies, our study first obtained differentially expressed mRNA and lncRNA using the edge R package and aberrant methylated genes using the limma R package, and then we filtered low expression genes and intersected mRNA and lncRNA expression data with DNA methylation data to obtain methylation-driven genes by using the MethylMix R package. The MethylMix (https://bioconductor.riken.jp/packages/3.1/bioc/html/MethylMix.html) is an algorithm implemented to integrate DNA methylation with RNA expression to identify methylation-driven genes in cancers [52]. In summary, MethylMix provides a tool that contributes to the analysis of methylation-driven lncRNAs and mRNAs in cancer studies from TCGA [17, 18]. However, the MethylMix focuses on identifying cis-regulatory effects of DNA methylation on gene expression and does not currently model trans-regulatory effects [18]. Further studies are needed to solve the multiple testing challenge on identifying trans-regulatory effects of DNA methylation on gene expression. Our study may provide a novel method for determining disease-specific prognostic biomarkers in LUAD and may play a significant role in predicting the diagnosis and prognosis of LUAD.

Our study subjects were retrieved from the TCGA database, which is a significant tool for analyzing prognostic biomarkers. It is not known whether our results are applicable to other groups. The predictive prognostic lncRNA and mRNA signature needs to be verified by molecular biologic experiments on clinical samples in future studies. Eventually, large-scale samples and experimental studies could validate the biological function of prognostic biomarkers in LUAD.

## Conclusion

In conclusion, our study identified methylation-driven mRNA and lncRNA by using bioinformatics analysis from the TCGA database. A Cox predictive model was performed to identify independent prognostic factors. Methylation and gene expression data combined with survival analysis was used to identify LUAD-specific, methylation-driven lncRNAs and mRNAs for predicting the diagnosis and prognosis of LUAD.

## Data Availability

All data are available from the sources listed in the manuscript—the TCGA data portal.

## Abbreviations

LncRNA: long noncoding RNA
LUAD: lung adenocarcinoma
GO: gene ontology
NSCLC: non-small-cell lung cancer
FC: fold change
FDR: false discovery rate
TCGA: The Cancer Genome Atlas Project
DAVID: The Database for Annotation, Visualization and Integrated Discovery
ROC: receiver operating curve
AUC: area under the ROC curve
